# Risks and complications of robot-assisted radical prostatectomy (RARP) in patients receiving antiplatelet and/or anticoagulant therapy: a retrospective cohort study in a single institute

**DOI:** 10.1007/s11701-020-01154-8

**Published:** 2020-10-12

**Authors:** Masashi Oshima, Satoshi Washino, Yuhki Nakamura, Tsuzumi Konishi, Kimitoshi Saito, Yoshiaki Arai, Tomoaki Miyagawa

**Affiliations:** Department of Urology, Jichi Medical University Saitama Medical Center, 1-847, Amanuma-cho, Saitama-shi, Saitama, 330-8503 Japan

**Keywords:** Prostatectomy, Anticoagulant, Antiplatelet, Heparin, Complication

## Abstract

The objective of the study was to evaluate the risk of bleeding complications in patients undergoing robot-assisted radical prostatectomy (RARP) while taking antiplatelet (AP) and/or anticoagulant (AC) agents. We analyzed the data of 334 patients undergoing RARP from May 2015 to May 2019. Patients were categorized into AP, AC, and control groups; the bleeding complications were compared among them. The end points were the estimated blood loss, decrease in hemoglobin level, and bleeding complications. The patient characteristics did not differ significantly among groups, with the exception of ASA scores, which were significantly higher in the AP and AC groups vs. the control group. The estimated blood loss and hemoglobin decrease were not significantly different between the AP and AC groups and the control group. The frequency of bleeding complications did not differ significantly between the AP and the control groups, but was significantly higher in the AC vs. the control group (4.3% in the AP and 23.5% in the AC group vs. 3.7% in the control group; *P* = 0.63 and *P* < 0.01, respectively). There was no significant difference in bleeding complications between the AP continuation (continuation of a single AP) and the AP interruption group or between the heparin bridging and the AC interruption group. All bleeding complications observed in the AC group occurred after resuming AC therapy. RARP can be performed safely with continuation of a single AP, and in patients taking ACs by interrupting these agents or via heparin bridging, without increasing intraoperative bleeding, whereas postoperative bleeding complications may increase after resuming ACs.

## Introduction

The aging of the population has led to an increase in cardiovascular conditions and in the intake of oral antiplatelet (AP) and/or anticoagulant (AC) drugs [1]. It is difficult for clinicians to handle patients taking these medications when a surgical intervention is considered. There is a dilemma between the bleeding risk if they are continued during the procedure and the thromboembolitic risk associated with their discontinuation. Most urological endoscopic and surgical procedures have a significant bleeding risk, and clinicians need to decide how to manage these agents in the perioperative period by considering the balance between the bleeding risk of the surgery and the thromboembolitic risk [2, 3].

In recent years, the use of robot-assisted radical prostatectomy (RARP) for prostate cancer has spread rapidly worldwide [4]. In addition, the number of patients taking APs and/or ACs has also increased. Therefore, it has become important to handle APs and/or ACs safely in patients undergoing RARP.

A panel consensus of the American Urological Association (AUA) and the International Consultation on Urological Disease (ICUD) was reached regarding the management of APs and ACs during the perioperative period of urological surgery [5]. This report stated that open retropubic radical prostatectomy (RRP) could be safely performed with continuation of perioperative APs. Conversely, there was no recommendation regarding the handling of perioperative ACs. Moreover, there was no statement on laparoscopic retropubic prostatectomy (LRP) or RARP in this report. RRP has a high risk of bleeding, while RARP is associated with a decreased risk of bleeding complications compared with RRP [6]. Therefore, the management of APs and/or ACs needs to be customized for RARP. However, few studies have evaluated the safety of RARP in patients receiving APs and/or ACs [7–12]. Currently, additional information on this subject is needed. In the present study, we retrospectively studied the safety of RARP in patients taking APs and/or ACs via comparison with a control group.

## Materials and methods

### Patients

We retrospectively analyzed the data of 334 patients who underwent RARP at the Jichi Medical University Saitama Medical Center from May 2015 to May 2019. All cases were operated using a da Vinci Si^®^ Surgical System (Intuitive Surgical Inc.) via a transperitoneal (conventional or Retzius-sparing RARP) or transretroperitoneal approach. A total of seven surgeons performed RARP.

**Fig.1:**
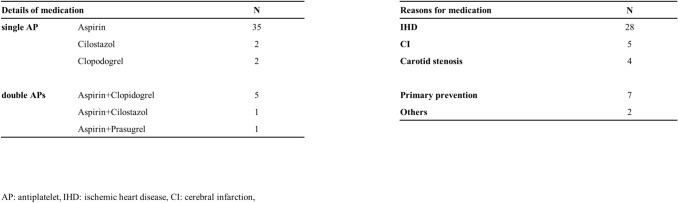
Categorization of the patients

Patients were categorized into the control group (A), the AP group (B), and the AC group (C). The AP group was sub-categorized into the AP continuation group (B1: patients with continuation of a single AP) and the AP interruption group (B2: patients with interrupted AP). The AC group was sub-categorized into the AC interruption group (C1: patients with interruption of AC) and the heparinization group (C2: patients switching from oral AC to heparin) (Fig. 1). A patient who was taking both AP and AC was categorized into the AC group. In the heparin-bridged patients, heparin was started at a dose of 10,000 U/day, adjusted to achieve an activated partial thromboplastin time that was 1.5–2.0 times that of the control value, and then discontinued 4–6 h before the surgery. In the interruption group, AP and/or AC drugs were re-started as soon as possible after the confirmation of the absence of major bleeding in the drain.

Indication for nerv-sparing, Retzius sparing, and lymph node dissection

The nerve-sparing technique is recommended in patients who have clinincal T1 and T2 prostate cancer with a PSA below 10 ng/dL, a Gleason Score < 7, and sexual function [13]. The Retzius-sparing technique results in a higher urinary continence rate after surgery, but might increase the rate of positive surgical margins [14]. The decision regarding the introduction of these techniques was made by the surgeon and the patients after detailed discussion. Lymph node dissection (LND) was performed only in cases with very high risk and cases with obvious lymphadenopathy in the pelvis, because the therapeutic values of LND remain controversial [15].

### Surgical procedure

In all patients, the VIO 300D system (ERBE Inc., soft coagulation mode) was used for intraoperative hemostasis. A hemostatic agent [TachoSil^®^ (CSL Behling Inc.) or Integran^®^ (Koken Inc.)] was also placed on the pelvic floor and/or the LND sites in patients who had minor bleeding at those locations. In patients who underwent nerve-sparing surgery, the neurovascular bundle was dissected athermally using clips.

### Postoperative management

In principle, patients were not allowed to walk on the day of surgery and started walking the next morning. Blood tests, including the assessment of hemoglobin levels, were performed routinely on the first postoperative day. The drainage tube on the pelvic floor was removed on the second or third postoperative day. The urethral catheter was removed on the 5th to 7th postoperative day, after cystography confirmed the absence of major leakage from vesicourethral anastomosis. Patients were discharged from the hospital on the day after cystography.

### Study end points

We compared the preoperative factors (patient characteristics), intraoperative factors (estimated blood loss, operation time, intraoperative blood transfusion, etc.), and postoperative factors (postoperative complications, drain placement period, hospitalization period, etc.) among the three groups.

The primary end points of this study were the estimated blood loss, the decrease in hemoglobin levels, and hemorrhagic complications after RARP among the control group (A), the AP group (B), and the AC group (C). Decreases in hemoglobin levels were determined using the following equation: [preoperative hemoglobin level—hemoglobin level on the first postoperative day]. Preoperative examinations were performed within 3 months before surgery.

### Statistical analysis

Statistical analysis was performed using the GraphPad Prism software version 8.0. Data were compared using Student’s *t* test, the Mann–Whitney *U* test, or the *χ*^2^ test. All data are presented as the mean and SD, unless otherwise indicated. For all statistical tests, significance was set at *P* < 0.05.

## Results

Data were collected from a total of 334 patients. One patient suffering from hemophilia A at surgery was excluded because hemophilia A itself is a substantial bleeding risk; therefore, 333 patients were analyzed. The cohort of 333 patients was categorized into the control (A; *n* = 270), AP (B; *n* = 46), and AC (C; *n* = 17) groups (Fig. 1).

Among the 46 patients in the AP group (B), 39 patients were taking single AP therapy and 7 patients were taking double AP therapy (DAPT). The details of the medications and the reason for taking AP agents are shown in Table 1. In the AP group, 7 patients interrupted AP before surgery and were not taking any AP in the perioperative period (AP interruption group: B1), while 39 patients continued to take one AP in the perioperative period (AP continuation group: B2) (Fig. 1). In patients taking single aspirin or cilostazol, these drugs were continued (*n* = 37), while in patients taking single clopidogrel, this drug was switched to aspirin 2 weeks before surgery and aspirin therapy was continued in the perioperative period (*n* = 2). In the seven patients taking DAPT, one AP (such as clopidogrel or cilostazol) was interrupted prior to the surgery, while aspirin alone was continued in the perioperative period.

**Table 1:** Details of the AP group (*n* = 46)

Single AP	Aspirin	35	IHD	28
	Cilostazol	2	CI	5
	Clopidogrel	2	Carotid stenosis	4
Double APs	Aspirin + Clopidogrel	5	Primary prevention	7
	Aspirin + Cilostazol	1	Others	2
	Aspirin + Prasugrel	1		

*AP* antiplatelet, *IHD *ischemic heart disease, *CI* cerebral infarction

Among the 17 patients in the AC group (C), 16 patients were taking single AC agents and 1 patient was taking AC and AP. ACs were interrupted in the perioperative period in seven patients, and both AC and AP were interrupted in the patient taking AP plus AC (AC interruption group: C1); in contrast, oral ACs were switched to heparin before surgery in ten patients (AC heparinization group: C2) (Fig. 1). The details of the drugs and the reasons for taking AC agents are shown in Table 2.

**Table 2:** Details of the AC group (*n* = 17)

Details of medication			*N*	Reasons for medication		*N*
Single AC (*n* = 16)	Warfarin		4	AC	AF	13
	DOAC	Apixaban	5		After valve replacement	2
		Dabigatran	3		Others	2
		Rivaroxaban	3			
		Edoxaban	1	AP	IHD	1
AP + AC (*n* = 1)	Aspirin + Dabigatran		1			

*AC* Anticoagulant, *AP* antiplatelet, *DOAC* direct oral anticoagulant, *IHD* ischemic heart disease, *AF* atrial fibrillation

### Patient characteristics

There was no significant difference in age, BMI, serum PSA level, clinical stage, and ISUP Gleason grade between the AP group (B) and the control group (A), or between the AC group (C) and the control group (A). About 30% of patients in the AP or AC group were classified as ASA risk III, while only 4% of patients in the control group were classified in this risk category (Table 3).

**Table 3:** Presurgical data

	Control Group	AP Group			AC Group			*p* value							
	(*n* = 270)	Interruption (*n* = 7)	Continuing (*n* = 39)	Total (*n* = 46)	Interruption (*n* = 7)	Heparinization (*n* = 10)	Total (*n* = 17)								
	A	B1	B2	B	C1	C2	C	A vs. B1	A vs. B2	A vs. B	B1 vs. B2	A vs. C1	A vs. C2	A vs. C	C1 vs. C2
Age mean ± SD	68.6 ± 6.0	67.9 ± 7.4	70.2 ± 4.4	69.8 ± 5.1	68.6 ± 2.9	72.4 ± 2.4	70.8 ± 3.2	0.74	0.12	0.2	0.28	0.99	0.05	0.13	0.01
Body mass index(kg/m^2^) mean ± SD	23.9 ± 2.8	24.0 ± 2.4	24.7 ± 3.2	24.6 ± 3.1	23.2 ± 3.0	22.9 ± 2.7	23.0 ± 2.8	0.9	0.09	0.11	0.6	0.52	0.25	0.2	0.82
Serum PSA (ng/mL) mean ± SD	10.6 ± 10.2	6.99 ± 2.58	14.9 ± 20.8	13.7 ± 19.4	9.47 ± 5.29	8.06 ± 3.09	8.6 ± 4.2	0.27	0.16	0.38	0.09	0.82	0.63	0.61	0.89
Clinical stage, *n* (%)															
cT1–T2	238 (88.2%)	7 (100%)	29 (74.4%)	36 (78.3%)	6 (85.6%)	9 (90%)	15 (88.2%)	> 0.99	0.03	0.1	0.32	0.59	> 0.99	> 0.99	> 0.99
cT3–T4	32 (11.8%)	0 (0%)	10 (25.6%)	10 (21.7%)	1 (14.3%)	1 (10.0%)	2 (11.8%)								
Biopsy ISUP grade, *n* (%)															
1	26 (9.6%)	4 (57.1%)	4 (10.2%)	8 (17.3%)	2 (28.6%)	2 (20.0%)	4 (23.5%)	< 0.01	0.56	0.3	0.04	0.06	0.62	0.28	0.45
2	114 (42.6%)	2 (28.6%)	20 (51.3%)	22 (47.8%)	0(0%)	4 (40.0%)	4 (23.5%)								
3	59 (21.9%)	1 (14.3%)	4 (10.2%)	5 (10.9%)	2 (28.6%)	2 (20.0%)	4 (23.5%)								
4	26 (9.6%)	0 (0%)	4 (10.2%)	4 (8.7%)	0 (0%)	1 (10.0%)	1 (5.9%)								
5	44 (16.3%)	0 (0%)	7 (17.9%)	7 (15.2%)	3 (42.9%)	4 (40.0%)	4 (23.5%)								
ASA physical status classification															
I	40 (15.3%)	0 (0%)	0 (0%)	0 (0%)	0 (0%)	1 (10.0%)	1 (5.9%)	0.01	< 0.01	< 0.01	> 0.99	0.44	< 0.01	< 0.01	0.04
II	219 (80.8%)	5 (71.4%)	27 (69.2%)	32 (69.6%)	7 (100%)	4 (40.0%)	11 (64.7%)								
III	11 (3.7%)	2 (28.6%)	12 (30.8%)	14 (30.4%)	0 (0%)	5 (50.0%)	5 (29.4%)								

*AP:* antiplatelet, AC: anticoagulant, *PSA* prostate-specific antigen, *ISUP* International Society of Urological Pathologists, *ASA* American Society of Anesthesiologists

#### Comparison of intraoperative data and surgical procedures (Table 4)

**Table 4:** Intrasurgical data

	Control group	AP group			AC group			*p* value							
	(*n* = 270)	Interruption (*n* = 7)	Continuing (*n* = 39)	Total (*n* = 46)	Interruption (*n* = 7)	Heparinization (*n* = 10)	Total (*n* = 17)								
	A	B1	B2	B	C1	C2	C	A vs. B1	A vs. B2	A vs B	B1 vs. B2	A vs. C1	A vs. C2	A vs. C	C1 vs. C2
Operation time (min) mean ± SD	229.3 ± 63.8	232.7 ± 30.5	230.6 ± 74.5	230.9 ± 69.7	206.9 ± 46.2	249.6 ± 75.1	232.0 ± 68.1	0.89	0.91	0.87	0.94	0.36	0.33	0.87	0.23
Console time (min) mean ± SD	187.9 ± 59.0	184.7 ± 26.9	186.0 ± 62.6	185.8 ± 58.6	167.0 ± 38.8	209.5 ± 68.5	192.0 ± 61.8	0.87	0.85	0.82	0.96	0.35	0.26	0.78	0.18
Estimated blood loss (ml) mean ± SD	101.6 ± 109.8	60.0 ± 46.3	109.0 ± 98.8	101.5 ± 94.4	116.4 ± 130.6	141.0 ± 141.9	130.9 ± 137.9	0.31	0.72	0.96	0.21	0.74	0.28	0.31	0.74
Hb reference (g/dL) mean ± SD	1.35 ± 0.90	1.23 ± 0.57	1.37 ± 0.92	1.35 ± 0.87	0.79 ± 1.94	1.04 ± 0.71	0.93 ± 1.36	0.73	0.89	0.99	0.7	0.12	0.29	0.08	0.73
Blood transfusion during surgery, *n* (%)	0 (0%)	0 (0%)	0 (0%)	0 (0%)	0 (0%)	0 (0%)	0 (0%)	NA							
Nerve sparing								0.04	0.14	0.13	0.05	0.55	0.32	0.06	0.54
None	186 (69.0%)	3 (42.9%)	32 (82.1%)	35 (76.1%)	5 (71.4%)	9 (90.0%)	14 (82.4%)								
One side	51 (18.8%)	4 (57.1%)	6 (15.4%)	10 (21.7%)	2 (28.6%)	1 (10.0%)	3 (17.6%)								
Both sides	33 (12.2%)	0 (0%)	1 (2.6%)	1 (2.2%)	0 (0%)	0 (0%)	0 (0%)								
Lymph node dissection, *n* (%)	30 (11.1%)	0 (0%)	5 (12.8%)	5 (10.9%)	0 (0%)	1 (10.0%)	1 (5.9%)	> 0.99	0.58	0.8	> 0.99	> 0.99	> 0.99	> 0.99	> 0.99

*AP* antiplatelet, *AC* anticoagulant, *Hb* hemoglobin, *NA* not assessed

The estimated blood loss [101.6 ml in A vs. 101.5 ml in B vs. 130.9 ml in C; *P* = 0.96 (A vs. B), *P* = 0.31 (A vs. C)]; hemoglobin decrease [1.35 g/dL in A vs. 1.35 g/dL in B vs. 0.93 g/dL in C; *P* = 0.99 (A vs. B), *P* = 0.08 (A vs. C)]; operative time; and console time during surgery were not different significantly between the AP (B) or the AC (C) group and the control group (A).

Moreover, the estimated blood loss (60.0 ml in B1 vs. 109.0 ml in B2; *P* = 0.21; and 116.4 ml in C1 vs. 141.0 ml in C2; *P* = 0.74) and hemoglobin decrease (1.23 g/dL in B1 vs. 1.37 g/dL in B2; *P* = 0.70; and 0.79 g/dL in C1 vs. 1.04 g/dL in C2; *P* = 0.73) were not significantly different between the AP continuation (B1) and the AP interruption (B2) groups, or between the AC interruption (C1) and the AC heparinization (C2) groups (Table 4).

#### Comparison of postoperative data (Table 5)

**Table 5:** Postsurgical data

	Control group	AP group			AC group			*p* value							
	(*n* = 270)	Interruption (*n* = 7)	Continuing (*n* = 39)	Total (*n* = 46)	Interruption (*n* = 7)	Heparinization (*n* = 10)	Total (*n* = 17)								
	A	B1	B2	B	C1	C2	C	A vs. B1	A vs. B2	A vs. B	B1 vs. B2	A vs. C1	A vs. C2	A vs. C	C1 vs. C2
Drain output on POD1 (ml) mean ± SD	124.8 ± 100.3	114.7 ± 38.6	145.6 ± 95.5	140.6 ± 89.9	89.0 ± 39.3	127.3 ± 153.5	111.5 ± 121.9	0.79	0.23	0.31	0.41	0.35	0.94	0.6	0.55
Catheter placement (days)															
Mean ± SD	9.0 ± 9.5	10.3 ± 11.3	9.3 ± 8.8	9.4 ± 9.2	11.7 ± 14.0	12.9 ± 9.1	12.4 ± 11.4	0.33	0.87	0.6	0.48	0.89	0.01	0.04	0.17
Median (IQR)	6 (6, 7)	6 (5, 6)	6 (6, 6)	6 (6, 6)	6 (6, 6)	8 (6, 19)	6 (6, 15)								
Hospitalization (days)															
Mean ± SD	7.5 ± 1.6	7.3 ± 1.2	7.6 ± 2.2	7.6 ± 2.1	6.9 ± 0.3	9.9 ± 2.7	8.6 ± 2.6	0.65	0.29	0.25	0.74	0.21	< 0.01	0.049	< 0.01
Median (IQR)	7 (7, 8)	7 (6, 9)	7 (7, 7)	7 (7, 7)	7 (7, 7)	9 (8, 12)	7 (7, 9)								
Blood transfusion after surgery, *n* (%)	0 (0%)	0 (0%)	0 (0%)	0 (0%)	0 (0%)	0 (0%)	0 (0%)	NA							
Bleeding complications*, *n* (%)															
Gd0	260 (96.3%)	7 (100%)	37 (94.9%)	44 (95.7%)	5 (71.4%)	8 (80.0%)	13 (76.5%)	0.87	0.55	0.63	> 0.99	< 0.01	0.02	< 0.01	0.46
Gd1	3 (1.1%)	0 (0%)	0 (0%)	0 (0%)	2 (28.6%)	1 (10.0%)	3 (17.6%)								
Gd2	7 (2.6%)	0 (0%)	2 (5.1%)	2 (4.3%)	0 (0%)	1 (10.0%)	1 (5.9%)								
Complications* during 90 days after surgery, *n* (%)															
Gd0	191 (70.5%)	4 (57.1%)	28 (71.8%)	32 (69.6%)	4 (57.1%)	5 (50.0%)	9 (52.9%)	0.74	0.86	0.78	0.67	0.12	0.39	0.08	0.44
Gd1	35 (12.9%)	1 (14.3%)	5 (12.8%)	6 (13.0%)	3 (42.9%)	3 (30.0%)	6 (35.3%)								
Gd2	39 (14.8%)	2 (28.6%)	6 (15.4%)	8 (17.4%)	0 (0%)	2 (20.0%)	2 (11.8%)								
Gd3	5 (1.8%)	0 (0%)	0 (0%)	0 (0%)	0 (0%)	0 (0%)	0 (0%)								
Gd4. 5	0 (0%)	0 (0%)	0 (0%)	0 (0%)	0 (0%)	0 (0%)	0 (0%)								

*AP* antiplatelet, *AC* anticoagulant, *POD* postoperative day, *Gd* grade, *NA* not assessed

*Complications according to Clavien–Dindo classification

There was no significant difference in any of the postoperative variables, including the frequency of bleeding complications, between the AP group (B) and the control group (A). The bleeding complications did not differ significantly between the AP interruption group (B1) and the AP continuation group (B2). Conversely, the frequency of bleeding complications was significantly higher in the AC group (C) compared with the control (A) (23.5% in C vs. 3.7% in A, *P* < 0.01), while there was no significant difference in bleeding complications between the AC interruption (C1) and the AC heparinization (C2) groups (28.6% in C1 vs. 20.0% in C2, *P* = 0.46) (Table 5). The durations of urethral catheter placement and hospitalization were also significantly longer in the AC group (C) compared with the control group (A).

The details of bleeding complications observed in four patients in the AC group (C) are listed in Table 6. Two patients were in the AC interruption group (C1) and two patients were in the heparinization group (C2). All four patients experienced hemorrhagic complications after resuming ACs. The complications in these cases were classified as grade 2 or less in the Clavien–Dindo classification. None of the patients developed thromboembolism.

**Table 6:** Details of cases with hemorrhagic complications

	Age	Intraoperative blood loss (ml)	NS	LND	Details of AC	Perioperative treatment of ACs	Resume of AC	Date of bleeding event	Detail of event	Clavien-Dindo classification	Treatment	Re-resume of AC
Case1	73	300	One side	None	Warfarin	Heparinization	POD3	POD4	Pelvic hematoma	Grade2	AC interruption	POD6
Case2	73	200	None	None	Dabigatran	Interruption	POD2	POD21	Gross hematuria	Grade1	Continuation	
Case3	68	100	None	None	Warfarin	Interruption	POD2	POD19	Gross hematuria, urinary retention	Grade1	Continuation	
Case4	72	100	None	None	Rivaroxaban	Heparinization	POD2	POD3	Progression of anemia	Grade1	AC interruption	POD6

*NS* nerve sparing, *LND* lymph node dissection, *AC* anticoagulant

## Discussion

The present study demonstrated that RARP was performed safely without increasing bleeding complications in patients taking APs with continuation of a single AP. Conversely, AC administration contributed to the increase in postoperative hemorrhagic complications, although all of them were grade 2 or less in the Clavien–Dindo classification.

A panel consensus of the AUA and ICUD was reached regarding how to treat APs and ACs during the perioperative period in urological surgery [4]. This report included the following statements: for patients on clopidogrel or aspirin for secondary stroke prevention, it is recommended to continue aspirin through the perioperative period; for patients with cardiac risk factors on low-dose aspirin alone, this can be continued in the perioperative period without increasing the risk of major bleeding; periprocedural management of direct oral anticoagulant (DOAC) therapy for patients with nonvalvular atrial fibrillation is stratified according to the procedural risk of bleeding and the urgency of the procedure; and the DOACs should be discontinued 2–5 days before elective surgery, with the timing dependent on the bleeding risk of the procedure, as discontinuation of DOAC can acutely increase the risk of stroke; therefore, bridging with another anticoagulant agent, such as heparin, is recommended. Conversely, it also stated that urological procedures with a high risk of bleeding, such as RRP, have been safely performed with bridging therapy in patients at higher risk for thromboembolic complications.

Few studies have explored the safety of AP use in RRP and LRP [12, 16], showing that APs did not increase the frequency of major bleeding complications. Several retrospective studies and one systematic review studied the safety of APs in RARP. According to these studies, continuation of aspirin is not correlated with an increased risk of perioperative surgical morbidity, blood loss, or hospitalization length, with the exception of a slightly higher transfusion rate in these patients (2.6% vs. 1.6%) [7–12]. In the present study, there were no cases of transfusion in either the AP or the control group, and the estimated blood loss was lower in both of the AP and the control group compared with that reported previously (Table 4). This might result from differences in the surgical procedures; e.g., we used a soft coagulation system and/or hemostatic agents for intraoperative hemostasis, which effectively stop bleeding from the prostatic or pelvic bed. Collectively, our results suggest that RARP can be performed safely without increasing major bleeding in patients taking APs with continuation of a single AP.

No studies have explored the safety of ACs in RRP or LRP, whereas one study has been published regarding RARP [17]. In that study, patients taking ACs had an increased operative time compared with the control group (189 vs. 170 min, *P* = 0.005) and hospital stay (1.4 vs. 1.1 days, *P* = 0.004), while the estimated blood loss (123.9 vs. 146.6 ml, *P* = 0.07), the 24 h change in hemoglobin level (2.2 vs. 2.3 g/dL, *P* = 0.44), and transfusion rates (6.7% vs. 1.7%, *P* = 0.07) were not significantly different between these groups. The comparison of the heparinization and interruption groups revealed a significantly greater transfusion rate (23% vs. 2%, *P* = 0.042) in the former; however, the complication and readmission rates were similar between these groups. One nonfatal thromboembolic event occurred in one patient in the AC interrupted group. In the present study, no patients received transfusions perioperatively in the AC group. Furthermore, there was no significant difference in operative time (229 vs. 232 min, *P* = 0.87), estimated blood loss (101.6 vs. 130.9 ml, *P* = 0.31), and 24 h change in hemoglobin (1.4 vs. 0.9 g/dL, *P* = 0.08) between the AC and the control groups (Table 5). However, postoperative bleeding complications (23.5% vs. 3.7%, *P* < 0.01) and urethral catheter placement duration (12.4 vs. 9.0 days, *P* = 0.04) were significantly increased in the AC vs. the control groups (Table 5), and all bleeding events occurred after resuming AC therapy (Table 6). Notably, there was no difference in the rate of bleeding complications between the heparinization and the AC interruption groups in the present study (Table 5), which implies that heparin bridging might be a safe option in RARP. However, the use of heparin bridging remains controversial. In a large RCT (BRIDGE trial), 1884 patients with atrial fibrillation who required interruption of warfarin for an invasive procedure were randomly assigned to receive bridging anticoagulation with low-molecular-weight heparin (Dalteparin) vs. a placebo [18]. The incidence of arterial thromboembolic events recorded 30 days after the procedure was similar in patients who received dalteparin and those who received the placebo (0.3% vs. 0.4%). The incidence of major bleeding was higher in those who received dalteparin (3.2% vs. 1.3%), although none of the bleeding events were fatal. Taken together, these findings suggest that RARP can be performed safely in patients taking ACs if these are interrupted or switched to heparin, while it is essential to check bleeding events carefully after resuming ACs. Heparin bridging is likely to be feasible in patients with high and very high thromboembolitic risks. More importantly, surgeons need to discuss these risks with the patients before surgery.

This study had several limitations, with its retrospective nature being its major drawback. Moreover, the number of patients included in the analysis was relatively small, especially in the AC group. Moreover, the handling of perioperative antithrombotic drugs was determined for each patient by the urologists and physicians; therefore, no clear standards were applied.

In conclusion, RARP can be performed safely with continuation of a single AP. Moreover, RARP can be performed safely in patients taking ACs by interrupting these agents or applying heparin bridging, without increasing the rate of intraoperative bleeding. However, postoperative bleeding complications may increase after resuming ACs.

## Data Availability

The datasets during and/or analyzed during the current study available from the corresponding author on reasonable request.
